# Ureteral Stone Mimics Appendicitis: A Point-of-care Ultrasound Case Report

**DOI:** 10.5811/cpcem.2020.7.48155

**Published:** 2020-10-05

**Authors:** Cindy Shavor, Justine Pagenhardt, YuanYuan Sun, Clara Kraft, Bradley End, Joseph Minardi

**Affiliations:** * West Virginia University School of Medicine, Department of Emergency Medicine, Morgantown, West Virginia; † West Virginia University School of Medicine, Department of Medical Education, Morgantown, West Virginia

**Keywords:** Point-of-care, syndromic approach, appendicitis, ureteral stone, case report

## Abstract

**Introduction:**

Abdominal pain is a common complaint in the emergency department. Point-of-care ultrasound (POCUS) is a rapid modality to evaluate for the etiology.

**Case Report:**

A teenage male presented with symptoms concerning for appendicitis. POCUS revealed a non-peristalsing, non-compressible, tubular structure containing an echogenic stone. This was determined to be a ureteral stone within a dilated ureter, not appendicitis.

**Conclusion:**

We propose a syndromic sonographic approach to right lower quadrant pain (RLQ) that includes the gallbladder, right kidney, bladder, and right adnexa, in addition to RLQ landmarks. This case emphasizes the value of such an approach to avoid diagnostic error.

## INTRODUCTION

Abdominal pain is a common presenting symptom for pediatric patients in the emergency department (ED). Pediatric patients or intellectually disabled patients can be challenging to care for due to their limited ability to explain symptoms, localize pain, and collaborate for a thorough exam.^1^ Even when a pediatric patient is able to localize pain, multiple diagnoses could be possible. A prime example is the complaint of right lower quadrant (RLQ) pain. Appendicitis is the most common surgical concern to rule out in the pediatric population. However, other diagnoses including intussusception, ovarian pathology, and inflammatory bowel disease such as Crohns must be considered.^2^,^3^ Selecting an imaging modality to safely and quickly evaluate patients is important.^4^ Ultrasound is a fast, inexpensive, and safe imaging technique that avoids radiation exposure in the pediatric patient.^5^,^6^

## CASE REPORT

A 17-year-old male presented to the ED with complaints of RLQ abdominal pain. He reported that the pain began a few hours prior to presentation to the ED. Associated symptoms included nausea and subjective fevers. Vital signs showed an elevated heart rate of 106 beats per minute and elevated blood pressure of 130/90 millimeters of mercury (mm Hg). He was afebrile without any increased work of breathing and normal oxygen saturation on room air. He was in no acute distress. Abdominal exam revealed positive bowel sounds and isolated RLQ tenderness without peritoneal signs. There was no suprapubic tenderness or costovertebral angle tenderness noted. The remainder of his examination was unremarkable.

**Image 1:**
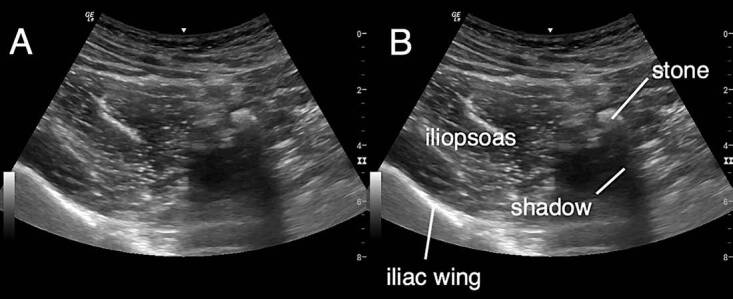
Ultrasound right lower quadrant (RLQ), short axis, curvilinear probe The landmarks of a RLQ ultrasound can be noted in short axis using the curvilinear probe with the iliac wing and iliopsoas muscle in view. Medial to the iliopsoas a hyperechoic focus representing a stone with acoustic shadowing is noted.

**Image 2:**
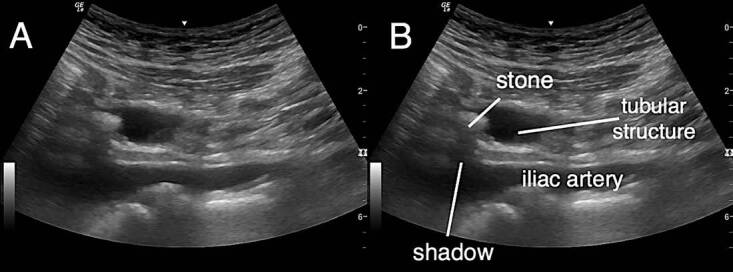
Ultrasound right lower quadrant, long axis, curvilinear probe A hyperechoic circular focus with acoustic shadowing is noted within a hypoechoic, non-peristalsing, non-compressible, tubular structure superior to the iliac vessels. A ureteral stone is identified as a hyperechoic structure, which demonstrates acoustic shadowing within the dilated ureter.

**Image 3:**
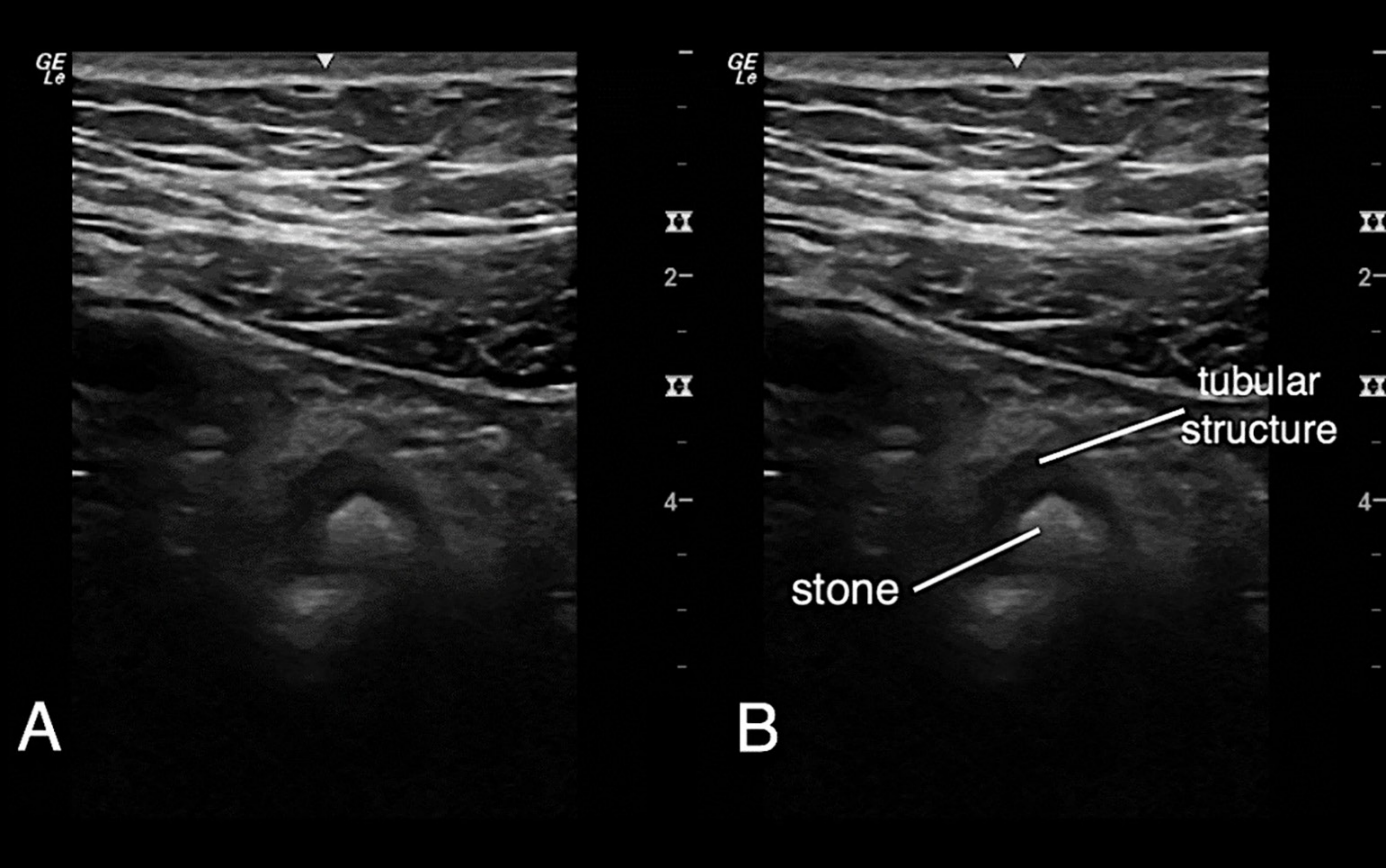
Ultrasound right lower quadrant, short axis, high frequency linear probe A hyperechoic circular object is noted within a hypoechoic tubular structure. The hyperechoic object is a ureteral stone noted within a dilated ureter.

Laboratory results showed a normal white blood cell count, normal C-reactive protein, and normal creatinine. A focused RLQ point-of-care ultrasound (POCUS) evaluating for appendicitis was performed. During the scan, a hyperechoic circular focus with acoustic shadowing was noted to be within a hypoechoic, non-peristalsing, non-compressible, tubular structure adjacent to the iliac vessels (Images 1–3). This was not the typical sonographic appearance of an infected appendix as there was no surrounding mesenteric fat stranding, free fluid, or hyperemia. Additionally, the walls of the tubular structure were thinner and did not have the alternating layered appearance typical of intestinal structures.

Because the clinical and sonographic findings were discordant, a computed tomography (CT) was performed confirming the suspicion that the finding in the RLQ was a large, 11-mm obstructing ureteral stone with hydroureter and hydronephrosis.

The patient was treated supportively with intravenous fluids and pain control and was admitted to the hospital. The patient underwent cystoscopy with placement of ureteral stent. He was discharged and later returned for stent replacement, lithotripsy, and stone extraction. The patient recovered without any noted complications.

## DISCUSSION

Ultrasound evaluation for RLQ pain is a common modality to diagnose appendicitis. Typical appearance of an inflamed appendix is a blind-ending, tubular, non-compressible structure without peristalsis that is greater than 6 mm in diameter. Other features include possible surrounding fluid, appendicolith, hyperemia of the bowel wall, inflamed surrounding mesenteric fat and lymphadenopathy.^1^ A dilated ureter is also a tubular, non-compressible structure without peristalsis. The ureter will typically appear as a thin-walled structure, whereas, the appendix has a multi-layered appearance. Often, an inflamed appendix will have a wall that appears thick due to the loss of definition of the layers. A ureteral stone may not look significantly different than an appendicolith. Because of these features, it would be possible for a physician to mistake these findings for those of acute appendicitis.

CPC-EM Capsule

What do we already know about this clinical entity?

*Point-of-care ultrasound (POCUS) is used to diagnose appendicitis and renal stones. Variations of pathology can mimic other syndromes leading to error*.

What makes this presentation of disease reportable?

*The uncommon presentation of a ureteral stone in the right lower quadrant could be easily mistaken for acute appendicitis leading to incorrect treatment*.

What is the major learning point?

*A ureteral stone may mimic the appearance of acute appendicitis. A systematic syndromic approach to POCUS can aid in rapid, accurate diagnosis*.

How might this improve emergency medicine practice?

*Recognizing the distinguishing factors between related pathologies on POCUS can limit medical errors*.

The incidence of renal stones has been increasing in pediatric patients.^7^ CT is becoming more commonplace as an evaluation modality to assess for renal stones in these patients. It is important to limit radiation exposure, especially in the pediatric population, as younger patients have a longer lifetime risk of malignancy related to radiation exposure through repeated examinations during their lives. CT is considered the gold standard for the diagnosis of renal stones in adults; however, it is advisable to employ strategies that limit this radiation exposure in children.^6^ Ultrasound also has the additional advantages including availability and lower cost. A study by Passerotti et al prospectively evaluated the ability of ultrasound to detect renal stones when compared to CT. In the study, ultrasound was found to have a 76% sensitivity and 100% specificity. The stones that were missed by ultrasound but identified by CT were on average 2.3 mm.^8^

While acute appendicitis is the most common surgical cause of RLQ pain, the spectrum of disease and differential diagnosis is quite broad and includes many etiologies that might be evaluated with a thoughtful, systematic, syndromic approach to the POCUS examination. Rather than focusing on a single organ or organ system, a syndromic POCUS is complaint based and may include multiple organ systems. The focused assessment with sonography in trauma (FAST) exam is likely the most common example. Another example may include evaluating the renal system as well as the aorta in an older patient with flank pain. In a review article, Chang, Schooler and Lee discuss the errors of diagnosing pathologic conditions in children who present with RLQ pain. They urged the consideration of etiologies beyond acute appendicitis: 1) congenital causes such as gastrointestinal duplication systems, Meckel’s diverticulum, and urachal abnormalities; 2) other inflammatory or infectious causes such as Crohn’s colitis, ulcerative colitis, Henoch-Schönlein purpura, mesenteric adenitis, omental infarction, and pelvic inflammatory disease; 3) neoplastic causes such as colonic polyps, ovarian masses, and lymphoma; and 4) other genitourinary causes such as pyelonephritis, ovarian torsion, ovarian cysts, endometriosis, and renal stones. Many of these conditions in the pediatric population are initially evaluated with ultrasound studies.^9^

A few case reports similarly discuss the initial evaluation of RLQ pain for appendicitis that revealed alternate pathology. One such case report discussed the use of ultrasound to diagnose a torn rectus abdominus muscle.^10^ The other case report described a patient with RLQ ultrasound that showed a tubular, non-peristalsing structure with a target appearance on short axis. This case was presumed to be appendicitis and the patient was taken for surgical intervention. Ultimately, the appendix was normal, and a torsed Meckel’s diverticulum was discovered. The RLQ ultrasound had identified the cause, which was misinterpreted as an inflamed appendix.^11^

There is value in discussing systematic syndromic ultrasound focused on a complaint-based evaluation rather than an organ-based approach. This necessitates evaluating multiple anatomic structures in the region of concern to systematically rule in or out pathological findings. This case was discussed with senior clinical ultrasound faculty at a joint case review focused on quality improvement in scanning technique and education. Anecdotally, another senior physician reported a similar case of hydroureter detected when initially scanning a pediatric patient with another diagnosis in mind. With this occurrence, we recommend scanning other potential areas of interest based on the patient’s clinical history, physical examination, and laterality of pain. For instance, this patient was having RLQ pain; scanning the gallbladder, right kidney, bladder, right adnexa, and right lower quadrant (Video) would have identified the right hydronephrosis and added further insight into the initial ultrasound finding in the RLQ of a hyperechoic object within a tubular structure.

## CONCLUSION

We present an ultrasound case of ureteral stone mimicking not only the symptoms, but also the sonographic findings of acute appendicitis. Nephrolithiasis, ureteral stones, and appendicitis are common diagnoses in the ED. With a thoughtful clinical approach and more systematic, syndromic POCUS, an accurate, timely diagnosis was made. A broad differential should be considered in the evaluation of RLQ pain, with a POCUS examination focused on high yield, relevant anatomical areas including multiple, right-sided organs including the gallbladder, right kidney, appendix, and right adnexa.

## Supplementary Information

Video Ultrasound right lower quadrant noting the various views and landmarks utilized to identify a ureteral stone within a dilated ureter.
